# Sharing “off-script”: A qualitative analysis of providers’ empathic self-disclosures during dignity therapy

**DOI:** 10.1017/S1478951524002098

**Published:** 2026-02-06

**Authors:** Emily L. Mroz, Tithi Amin, Sheri Kittelson, Mary Kate Koch, Alyssa Crowe, Susan Bluck, Joshua Hauser, George F. Handzo, Diana J. Wilkie, Carma L. Bylund

**Affiliations:** 1 Section of Geriatrics, Department of Internal Medicine, Yale School of Medicine, New Haven, CT, USA; 2 Nell Hodgson Woodruff School of Nursing, Emory University, Atlanta, GA, USA; 3 Department of Health Outcomes and Biomedical Informatics, University of Florida College of Medicine, Gainesville, FL, USA; 4 Division of Palliative Care, Department of Medicine, University of Florida, Atlant, FL, USA; 5 Department of Psychology, University of Florida, Gainesville, FL, USA; 6 Department of Psychology, Gonzaga University, Spokane, WA, USA; 7 Feinberg School of Medicine, Northwestern University, Evanston, IL, USA; 8 Jesse Brown VA Medical Center, Chicago, IL, USA; 9 Health Care Chaplaincy Network, NY, USA; 10 Department of Biobehavioral Nursing Science, College of Nursing, University of Florida, Gainesville, FL, USA

**Keywords:** Empathic communication, psycho-oncology, life review, patient–provider communication, nurse-led intervention, chaplain-led intervention

## Abstract

**Objectives:**

Healthcare provider self-disclosures are common although sometimes controversial. Providers have unique opportunities to self-disclose for the purpose of conveying empathic concern during Dignity Therapy sessions. We examine the topics of empathic self-disclosures (ESDs) during Dignity Therapy sessions.

**Methods:**

We analyzed 203 audio-recorded, transcribed Dignity Therapy sessions from a stepped-wedge, randomized trial of Dignity Therapy led by 14 nurses and chaplains in outpatient palliative care. We extracted 117 ESDs across sessions and applied thematic analysis guided by the constant comparative method to generate ESD topic themes and properties.

**Results:**

Providers disclosed ESDs referring to topics of *Relationships and Family, Personal Experiences and Characteristics, Cohort Communalities, Location and Geography*, and *Values.* Though each provider led multiple Dignity Therapy sessions in this dataset, providers rarely disclosed the same information to more than one patient. Some disclosures subtly shifted the patient’s life review. Providers often acknowledged patients that their self-disclosures were not prescribed elements of Dignity Therapy sessions.

**Significance of results:**

Providers engage in ESD across a range of personal topics in a Dignity Therapy context. Some ESD topics overlapped with those considered appropriate in existing health communication literature. Other topics involved complex or underexamined types of disclosures. While self-disclosures appear to be made with empathic intent, providers undermined the impact of some ESDs by portraying them as unprescribed components of the conversation. More research is needed to assess the positive and negative impacts of ESDs during Dignity Therapy and to support augmentation of Dignity Therapy training protocols to account for providers’ ESDs.

Adult patients living with advanced cancer can benefit from palliative care practices delivered by health-care providers that address their psychospiritual concerns (Institute of Medicine 2015). Such practices often involve patients sharing intimate personal details, autobiographical memories, and complex emotions as they process their diagnosis and prognosis, reflect on their medical care experiences, and consider their priorities for living well with cancer (Stanley and Hurst 2011; Teo et al. 2019). Given the gravity of this social sharing context, providers’ empathic responses to patients have the capacity to bolster rapport, shape conversation dynamics, and even influence the outcomes of care (Johnston et al. 2015; Zink et al. 2017). Dignity Therapy is one practice that has shown marked success in improving patient outcomes for depression, anxiety, and existential concerns (Martínez et al. 2017). Although not an explicit part of the protocol, during Dignity Therapy, providers address patients using a variety of empathic responses, including the disclosure of shared experiences. Through this disclosure, the provider relates to the patient by describing their own personal experience, value, or feeling in response to the patient that reflects or complements what the patient disclosed (Bylund et al. 2022). Little is known about these types of disclosures made in palliative care contexts, including during Dignity Therapy. Therefore, the impact these disclosures may have on patient communication and practice outcomes is unknown (Mann 2018). The current study provides a foundation for addressing this gap by characterizing topics of providers’ empathic self-disclosures (ESDs) during Dignity Therapy.

Dignity Therapy has become a widespread, promising palliative care practice for cancer patients partly because it is a brief activity with lasting benefits for the patient, their family members, and close others (Fitchett et al. 2015). During Dignity Therapy, providers use guiding questions to engage patients in a life review that facilitates patients’ recounting of significant life memories, values, social relationships, and components of the legacy they wish to leave (Chochinov et al. 2005). Providers are trained to enrich and sustain this life review process by probing for more insight from patients and approaching each session with compassion and curiosity. In this context, providers naturally offer empathic responses to patients’ descriptions of emotional, challenging, or meaningful memories and beliefs. Sometimes, these responses are delivered in the form of providers’ self-disclosures of their own related emotional, challenging, or meaningful memories and beliefs (Bylund et al. 2022) and, as such, are considered by our team to be shared for the purpose of *ESD* (Crowe et al. 2024). Though ESD was not built into the Dignity Therapy process explicitly, because of the frequency of such disclosures (Bylund et al. 2022), this communication strategy may shape the Dignity Therapy process.

Self-disclosure has been described as a somewhat controversial, though common (Henretty and Levitt 2010; Mann 2018), provider communication behavior in some medical and therapeutic settings (Allen and Arroll 2015; Berg et al. 2020), including oncology care settings (McDaniel et al. 2021). On one hand, self-disclosure is seen as highly empathic (Bylund and Makoul 2005): in experimental (Kadji and Mast 2021) and specialty clinical settings (Beach et al. 2004; Zink et al. 2017), providers who offer self-disclosures during clinical discussions are seen as more caring, friendly, and warm as compared to providers who do not self-disclose. Alternatively, self-disclosures may be seen as tangential or disruptive to clinical discussions, potentially pulling the focus of the encounter away from the patient or derailing the patient’s own disclosures (Arroll and Allen 2015; McDaniel et al. 2007, 2021). Perhaps due to these concerns (Rousseau 2009), providers in specialty palliative care contexts have been shown to rarely engage in self-disclosure despite frequently engaging in other forms of empathic communication (Mroz et al. 2022, 2023). Some researchers have suggested that the empathic impact of providers’ self-disclosures may depend on the *types* of disclosures made in terms of the topics addressed. Allen and Arroll (2015), for example, developed a list of hypothetical self-disclosure topics that providers could utilize and found that providers’ comfort with self-disclosure varied across topics. Still, researchers have not empirically developed sets of context-specific self-disclosure topics, limiting investigation of the impacts of such disclosures within clinical settings, including Dignity Therapy contexts.

## Current study

This study is a secondary data analysis that was conducted as part of a multisite, stepped-wedge, randomized trial examining the use of Dignity Therapy to improve seriously ill cancer patients’ sense of dignity and other psychospiritual outcomes (Kittelson et al. 2019). As a component of that larger project, we examined providers’ communication. This allowed for a systematic examination of ESD in this context. In this, the first study of empathic provider self-disclosure (ESD) in Dignity Therapy sessions, our goal was to characterize the types of ESDs made by providers, in terms of disclosure topics, by addressing the research question: *when Dignity Therapy providers engage in self-disclosure, what types of disclosures do they make?* Our secondary aim was to enrich our characterization of ESD topics by describing common patterns in ESD during these sessions.

## Method

The University of Florida Institutional Review Board (IRB201601190) approved all research activities. All study procedures for the original trial were approved by the Institutional Review Boards at all study sites. We followed relevant guidelines from the COnsolidated criteria for REporting Qualitative research (COREQ; Tong et al. 2007) to describe our method and results, and the ICMJE Recommendations for the Conducting, Reporting, Editing, and Publications of Scholarly Work in Medical Journals to write and format the manuscript.

### Participants

This study involved analysis of transcribed Dignity Therapy sessions between trained Dignity Therapy providers and 203 middle-aged and older adult (*M*_age_ = 65; *SD* = 7; range = 55–87) patients living with cancer. Interested readers can learn more about the composition of the sample of Dignity Therapy patients elsewhere (Wilkie et al. 2023). Across study sites, Dignity Therapy sessions were conducted by 14 trained providers, the majority of whom were women (*n* = 10). All providers were either nurses (*n* = 5) or chaplains (*n* = 9). Dignity Therapy providers completed an average of 14.5 sessions each (range = 2–45). To protect the privacy of this small group of providers, further identifying information (e.g., age, race, ethnicity) is not reported here.

### Procedure

Data were collected between 2018 and 2021. The processes of involving seriously ill patients in Dignity Therapy and training Dignity Therapy providers is described in foundational works (Chochinov et al. 2005; Schoppee et al. 2022b) and in the protocol for the parent, multisite cluster randomized trial (Kittelson et al. 2019). Briefly, cancer patients were first recruited based on pre-determined eligibility criteria, including that they were (1) diagnosed with cancer, (2) receiving outpatient palliative care, and (3) 55 years or older. Patients were then randomized to the intervention condition based on a stepped-wedge timeline and provided written informed consent to participate in the trial.

Patients engaged with the Dignity Therapy provider in person or remotely, via videoconference. These audio-recorded and transcribed sessions were structured by guiding questions (Hack et al. 2010), and Dignity Therapy providers demonstrated practice fidelity to a moderate degree (Schoppee et al. 2022a). Important to the current study, all Dignity Therapy providers were trained to follow the guiding questions and fidelity benchmarks but also encouraged to structure the session with additional probing questions, reflections, or other communication techniques to facilitate a rich and comfortable life review. Thus, specific interjections from providers during sessions, including ESDs, were neither defined as standards for Dignity Therapy nor discouraged during training. Dignity Therapy providers or their institutions received $150 for each patient to which the Dignity Therapy was provided. Dignity Therapy sessions lasted an average of 42.60 minutes (*SD* = 10.50; range = 23–57) (Al Yacoub et al. 2023).

As we were interested in the provider’s ESDs, only those transcripts that involved such disclosures from providers were included in the analysis. A team of coders was assembled to conduct interaction analysis (Bylund and Makoul 2005; Bylund et al. 2022) to assess all transcriptions and accompanying audio files of Dignity Therapy sessions for the presence or absence of ESDs. That team was led by this study’s senior author (CLB; described further below) and a postdoctoral fellow with content analysis expertise (MKK). The team included 2 research assistant coders who had college education. The senior author first developed a refined version of the Empathic Communication Coding System (Bylund and Makoul 2005) which was modified for use with Dignity Therapy interview data (Bylund et al. 2022). Importantly, ESDs were considered present in transcript excerpts when the provider disclosed something substantial and personal about themselves in response to something the patient shared. The team finalized and applied guidelines for identifying ESDs in Dignity Therapy interview transcripts (see Supplemental File 1). The coding team had excellent reliability (κ = .80, ICC = .80). Pairs of independent coders each coded transcripts for ESDs and resolved discrepancies by consensus. A third of the Dignity Therapy sessions in our sample (37.25%, *n* = 76) included at least 1 ESD. ESDs could be detected more than once in a patient transcript, and often were: 23.68% (*n* = 18) of transcripts with 1 ESD involved a second ESD or more. As such, the final sample for qualitative analysis consisted of 117 ESD excerpts from Dignity Therapy providers. For information on the distribution of ESDs by provider characteristics (e.g., discipline, gender), see Crowe et al. (2024).

### Data analysis

We employed thematic analysis using a constant comparative approach (Corbin and Strauss 2008; Glaser and Strauss 1967), which guides iterative comparing of emergent concepts to identify topic-level thematic patterns supported by specific properties. This approach was ideal because it emphasizes revisiting the data at each procedural step to verify themes and properties accurately reflect what providers shared. We assembled a diverse analysis team (ELM, TA, SK, MKK, and CLB) to ensure multiple perspectives guided the analytic process: members consisted of 5 female researchers in different career stages spanning different disciplines, all of whom had interest in the topic because of its relevance to their larger programs of research. Specifically, as the time of data analysis, ELM held a PhD in psychology with additional training in gerontology; she was a postdoctoral fellow with expertise in qualitative methods. TA was a research assistant and study coordinator; she previously completed some coursework in psychology, public health, and health professions as an undergraduate student. SK held an MD and board certification in both family and palliative medicine. She was a full professor and Division Chief in the Department of Medicine with experience in cancer research and quality outcomes. MKK held a PhD in developmental psychology; she was a postdoctoral fellow with expertise in narrative analysis. CLB held a PhD in Communication Studies; she was a professor of Health Outcomes and Biomedical Informatics with expertise in empathic communication. The analysis team members were all familiar with each other prior engaging in this study but had no interaction with study participants. We conducted analyses using Atlas.ti software.

The team followed the 6 standard steps of thematic analysis (Terry and Hayfield 2021) that harmonized with benchmarks of the constant comparative approach, beginning with familiarizing themselves with the data by reviewing the transcript excerpts, recording memos, and discussing initial ideas for codes as a group. Following a process of open coding conducted by the whole analysis team, 2 team members (TA and CLB) took the open codes and, through another in-depth review of the data, collapsed the codes into themes. These candidate themes were used to develop the first iteration of a codebook, which was also scaffolded by information about hypothetical self-disclosure topics previously discussed in the literature (Allen and Arroll 2015) and memos describing observations from the study team. The analysis team reviewed this codebook version, provided suggestions for refining the themes, and then presented candidate themes to other study team members (i.e., including SB, JH, and GH). Using the data, the analysis team then generated ideas for properties (i.e., smaller units of meaning to enrich description of themes). The team employed relevant criteria for establishing thematic saturation (i.e., recurrence and forcefulness) throughout the process (Owen 1984).

Two team members (TA and ELM) then performed axial coding, revisiting the data to refine candidate properties within each theme (Williams and Moser 2019) and thicken descriptions of themes and properties in the codebook. Often, transcript excerpts represented more than 1 property; team members took care to detect frequent overlaps of properties, using these instances to refine the codebook. When relevant, property names were generated *in vivo*, meaning we used a provider’s own words as a representative label. The new codebook was reviewed in tandem with the data once more by all authors. We took steps to uphold study rigor through supporting *auditability* (i.e., by retaining all records, including codebook versions and transcript excerpts), *confirmability* (i.e., by using Atlas.ti to document systematic application of themes and properties and accrual of exemplars), and *dependability* (i.e., maintaining regular team touchpoints, comparing transcript excerpts across all data collection locations to ensure thematic consistency). Participants did not provide feedback on study findings.

## Results

**Figure 1.**
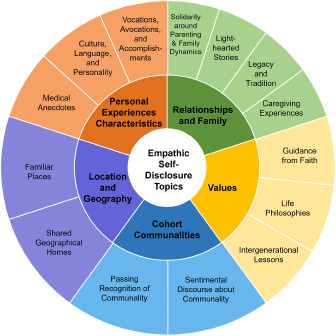
Empathic self-disclosure topic themes and properties.

Thematic analysis generated 5 ESD themes supported by 14 properties. Specifically, Dignity Therapy providers made disclosures about their *Relationships and Family, Personal Experiences and Characteristics, Cohort Communalities, Location and Geography*, and *Values* (Fig. 1). Below, we describe each self-disclosure topic theme and associated properties, then other essential elements of these ESDs noted during qualitative coding. Exemplar quotations are modified to protect provider confidentiality.

### Relationships and Family

Providers empathically self-disclosed information about their *Relationships and Family*, describing experiences with, traits of, and social connections between family members, friends, close others, neighbors, pets, and acquaintances. This theme was further characterized by 4 properties. The first, *Lighthearted Stories*, entails providers sharing personal narratives about happy, positive, or quirky experiences with family members or close others. These included surprising events or references to similarities between the patient and the provider’s family member when something the patient said or did reminded them of a family member, such as this narrative from a provider, “I had the same thing … a [child] that [was] with his [partner for many years]. One day he just called me up and said, ‘I think we wanna have a baby …’ They got a little [child of their own] now, and everybody’s happy.”

Next, providers shared expressions of *Solidarity around Parenting and Family Dynamics*, validating patients’ family challenges, the complexities of parenting and being parented, and heredity or family health behaviors through sharing their own experiences or perspectives. For example, when a patient shared that they believed their ‘odd’ personality came from their mother’s parenting, 1 provider shared, “I don’t wanna sidetrack us, so I’ll just make a comment. I think that unwittingly and unintentionally parents do pass things on..these things get absorbed … we can begin to shift it and say, ‘I don’t need to be like this now.’”

Providers also disclosed their views on the value of leaving a legacy for loved ones, upholding traditions, or maintaining connections with the deceased across generations, captured in the property *Legacy and Tradition*; 1 provider shared, “I want [my family] to be able to have a few things that they can hang their hat on when they think about me.” At times, these perspectives were disclosed when the provider was emphasizing the value and outcomes of the Dignity Therapy process itself, such as when this provider shared, “There’s a lot of stories I asked my [family member] to journal and write down before he died... are there any things, maybe, you want your family to know about you that are important?”

Finally, providers disclosed personal *Caregiving Experiences*, where they provided long-term support for a family member or friend living with chronic illness or disability, or in some cases, described observing family or friends offer caregiving support to others. Sometimes, the provider disclosed these as part of characterizing insights gained from witnessing others’ illness experiences, as mentioned by this provider, “We never had to have my [family member] in a hospital bed. He was in a double bed and everybody could come lay … with him.… I know those things are hard to think about, but it sounds like y’all are moving in that direction.”

### Personal Experiences and Characteristics

Providers self-disclosed through sharing about their own personal pastimes, lifestyles, or self-qualities, including describing their core characteristics or prominent personal attributes. Three properties further characterized this. The first, *Vocations, Avocations, and Accomplishments*, involved sharing about hobbies, talents, pastimes, recognitions, or occupations the individual engaged in. These were sometimes shared in response to patients’ disclosures of similar activities or careers. For example, when a patient expressed concern that they weren’t a good listener as a parent, 1 provider shared a self-disclosure related to their *Vocation*, which was similar to the patient’s vocation, and described how people with certain callings, “… don’t make great parents.” In other cases, the provider shared what has brought them joy or meaning in life. For instance, when the patient referenced spending time outside, a provider shared, “I love gardening and stuff. I try and do it in the evenings when it’s a little bit cooler,” although this was not a hobby shared by the patient, who replied, “I used to do it. I just can’t handle the heat anymore, [and] too many bugs out where I live.”

Providers also disclosed information about their *Culture, Language, and Personality*, including culture-based preferences, customs, emotional dispositions or central personality traits. This included showcasing personality traits by describing times in their lives when their personalities (e.g., competitive, self-assured, introverted) shone. This also involved disclosing genuine emotional responses to aspects of patients’ life reviews. On hearing about a patient’s family member’s health challenges, a provider said, “Whoa, well, I wanna say just parenthetically, I feel such sympathy for what your family went through.”

Finally, providers disclosed *Medical Anecdotes*, which included medical concerns or encounters, sometimes in response to the patient’s description of their own medical experiences. For example, in response to a patient’s discussion of health condition heritability, 1 provider said, “If it’s anything, my mother’s Rh-negative and I’m positive, so I’m sort of familiar with the phenomenon.” Some *Medical Anecdotes* pertained to someone a provider knew, rather than themselves; for example, 1 provider disclosed, “Wow. I hate that. I have a friend that has the [same type of cancer] and he’s had a lot cut out, so I hear ya.”

### Cohort Communalities

Providers disclosed knowledge about the ways things worked or what occurred during an era or period of time, often to provide an example of something they had in common with the patient or to indicate that the provider and patient were from a shared developmental cohort. Often, these disclosures were shared with a sense of nostalgia or fondness for the past or evoked fondness or nostalgia in the patient’s reply. By disclosing these details about themselves, these providers acknowledged that they could understand what patients experienced, contributed to, or struggled with during historical periods to the present day. For example, when 1 patient shared, “I played with dolls a long time,” the provider shared, “That’s all right [laughter]. I think I did, too, that’s what we did back then.”

These self-disclosures were structured in 2 ways. The first, *Passing Recognition of Communalities*, included brief acknowledgment of a communality without redirecting the conversation or encouraging a lengthy response from the patient. For example, a patient described the Kmart “Blue Light special,” and the provider spontaneously shared, “Oh, the Blue Light Special. I remember that,” after which the conversation continued without sidetracking. The second property involved *Sentimental Discourse about Communalities*, where the provider offered enough detail or passion about the communality that the disclosure redirected the conversation or prompted a more in-depth patient response. For example, 1 provider asked, “How old are you?” then shared, “yeah, I’m close to your age,” at which point the patient shared, “you know some of the transformations that we went through, different parts of life. You should remember the good times we went through …”

### Location and Geography

Providers made self-disclosures that revealed that they had lived in, visited, or knew something about a location the patient mentioned in their interview. These were characterized by 2 properties. The first a property named with the *in vivo* quote *“Familiar Places”* included places the provider was aware of, had traveled to, had family ties to, or otherwise appreciated but had never lived in. These disclosures sometimes involved the provider reflecting on how interesting or meaningful it was that these *“Familiar Places”* overlapped between the patient and provider; as mentioned by 1 provider, “I’m laughing ‘cause I hear all these connections since both my parents went [to that school].. these are all familiar places. Forgive me for saying that.” In addition, providers self-disclosed that they had *Shared Geographical Homes* in instances where they and the patient had called the same geographical place “home” in the past or present. Here, providers often emphasized a sense of shared community with the patient, such as when this provider shared, “I live around the corner from [your church]. You’ve had a great reputation. I don’t know you, but I have heard of you through the years. The legacy you have built to that church runs through the neighborhood.”

### Values

Finally, providers shared their *Values*, including personal missions, philosophies, lessons, or group affiliations that guided their everyday behaviors or offered a sense of personal comfort. These were sometimes disclosed to underscore the importance of patients’ described missions, philosophies, lessons, or group affiliations. Other times, this involved the provider disclosing how much they enjoyed being part of Dignity Therapy sessions because they appreciated hearing the *Values* the patients shared during sessions. This theme was defined by 3 properties. The first, *Guidance from Faith*, involved the provider disclosing lessons learned from religion or the value of religious ritual. This involved the provider disclosing the value they ascribed to prayer and disclosing that they intended to pray for the patient, as shared by this provider, “Know that you’re held in prayer. I know prayer is something that’s important for you. With your permission, I’ll continue to hold you in prayer.”

*Values* also included *Life Philosophies* disclosed by the provider, such as their take on living well, responding to others, perspective-taking and navigating challenges, making peace and healing, or balancing multiple goals or needs in life. For example, when a patient was describing parenting, 1 provider shared their strategy for giving gentle redirection, or “… the kind of discipline … so that people want to be with them,” then proceeded to reflect this anecdote back to the patient’s story, saying, “it sounds like people want to be with you. It does. It sounds like these young men come to you ‘cause they see you do good work.” *Life Philosophies* also included the provider rephrasing the patient’s philosophy using their own words, possibly to convey their own understanding of or agreement with the patient’s philosophy. Finally, providers shared *Intergenerational Lessons*, including lessons or insights about life passed up or down generations within their own families or between mentors and mentees. This sometimes involved learning how to raise children well; for example, 1 provider shared that their family member used to give advice about parenting that they have now adopted themselves.

### Patterns in provider ESD

ESDs often fit with multiple properties, indicating that properties are interrelated; in other words, some properties are conveyed through the sharing of information characterized by other properties. For example, embodied viewpoints characterized as *Life Philosophies* or *Intergenerational Lessons* can be conveyed through *Lighthearted Stories. Guidance from Faith* can also inform goals for *Legacy and Tradition* articulated by providers. ESDs varied across encounters, such that providers rarely disclosed the same personal information to more than 1 patient. In turn, reactions from patients to providers’ ESDs differed qualitatively across disclosures. Some disclosures served mainly to point out common ground between the patient and provider (e.g., a provider sharing that they can relate to introverted tendencies because they are also an introvert), and others subtly shifted or enriched the patient’s life review (e.g., a provider sharing that she has friends who just completed a Birthright trip to Israel, prompting the patient to describe their own experiences visiting Israel years ago through a program run by the Israeli army).

Providers at times acknowledged that their self-disclosures were not prescribed elements of Dignity Therapy sessions, identifying them in these instances as unscripted, impromptu, or impulsive contributions to the conversations, as mentioned by a provider who stated, “I’ll just say this kind of off-script, but you’re talking to the [parent] of a child with [a developmental disability], so I appreciate your work,” and as seen in exemplars above. Finally, we note that, on several occasions, providers indicated to the patient that they would end the recording before disclosing additional information about themselves to the patient informally, outside of the Dignity Therapy session. These indications often appeared to precede sharing more information related to *Location and Geography* (e.g., “I’ve so enjoyed this. Thank you … I didn’t tell you this, I grew up in [city], just so you know … this was an anecdote I was gonna mention to you … oh, it’s still recording. Let me turn the recording off.”).

## Discussion

We found that providers engaged in ESDs spanning a range of topics, from describing their own families or geographical homes to articulating their life philosophies and accomplishments. Using this large dataset, we found that specific ESDs were rarely repeated by providers, implying that providers did not “queue up” the same disclosures to covey empathic concern across multiple patient sessions. Instead, providers appear to fit ESDs to each discussion, choosing which information to share based on the discussion context. This research provides context for considering the effects of providers’ ESDs during Dignity Therapy and other life review practices offered to patients living with advanced cancer.

While conveying empathic concern for the patient, in some cases, providers’ ESDs may have served the additional, related function of rapport building: closing emotional gaps between patient and provider through description of common ground, or portraying the provider as relaxed and open (English et al. 2023). In other cases, providers connected their ESDs more squarely to the purpose of the Dignity Therapy session (Vuksanovic et al. 2017), potentially encouraging deeper engagement with the practice. For example, providers disclosed their own observations of the value of preserving *Legacy and Tradition* or documenting *Intergenerational Lessons.* In some cases, even brief ESDs from providers revealed a generous amount of information about the provider, offering patients opportunities to choose to explore these disclosures more deeply by asking follow-up questions or building on the provider’s train of thought. In other cases, ESDs manifested as brief interjections that did not seem to change the flow of conversation on the surface but may have influenced patients’ experiences in other ways.

We note that the ESD topics generated from our analysis partially map onto disclosure topics described by other health communication researchers (Allen and Arroll 2015). ESD themes regarding providers’ *Relationships and Family, Personal Experiences and Characteristics*, and *Values* generally map onto disclosure types previously posited by researchers as arising during primary care appointments. While, overall, general practice providers have rated themselves as comfortable disclosing information on these 3 topics in clinical encounters, some of our specific properties within these topics (e.g., *Medical Anecdotes, Solidarity around Parenting and Family Dynamics*) were rated as less appropriate to share in primary care appointments (Allen and Arroll 2015). In comparison, in the context of supporting patients with advanced cancer to complete dignity-preserving life reviews, providers may feel more inclined to share more serious or emotionally complex ESDs about their medical experiences or family challenges in addition to comparatively lighter or casual disclosures. During sessions, providers may feel that more complex or serious disclosures invite patients to expand upon their own challenges, offering opportunities for meaning-making processes that are considered central to Dignity Therapy (Bluck et al. 2022; Kadji and Mast 2021). While this desirable outcome may occur for some patients, we recognize that other patients may be baffled or frustrated by these types of disclosures, stymying rather than supporting meaning-making and other important psychological processes.

Still, other topics generated from our analysis have not been hypothesized as appropriate *or* inappropriate by researchers: disclosures around *Location and Geography* and *Cohort Communalities*, common in these Dignity Therapy transcripts, are less often addressed in other health communication literature. We speculate that both ESD types may be particularly beneficial to promote a sense of the provider’s acceptance of the patient and their life story (English et al. 2023; Sivell et al. 2019): when providers disclose that they, too, have lived in the same place or through the same historical period, they may mollify any power imbalance felt between the patient and provider. However, we also acknowledge that for some patients, these disclosures may unintentionally derail the conversation, for example, by shifting the focus to aspects of the life story that the patient had not meant to focus on or causing them to feel like they are required to continue to share personal details related to the provider’s disclosure.

While ESDs were common and varied in these Dignity Therapy transcripts, we recognize that they appear to have been used strategically. For example, in many cases, they were portrayed by providers as ancillary to the communication (e.g., as anecdotes that were “off-script,” “sidetracks,” or “parentheticals”). Dignity Therapy providers may have communicated this way to provide what they consider to be a highly empathic response to patients (Bylund and Makoul 2005) while still presenting their disclosure as supplemental so as to not steer patients too far off their own course. In addition, we note that ESDs only manifested in about a third of transcripts, presumably when the provider felt that the disclosure would encourage or enrich the patient’s life review. At the same time, presenting ESDs as ancillary may have the opposite effect, taking the patient out of the moment as they are reminded that they are participating in a scripted discussion that only veered “off-script” momentarily.

To best capitalize on the benefits of ESDs during Dignity Therapy while avoiding some pitfalls, we consider that our study’s findings, along with additional research, may be used to augment Dignity Therapy training programs. Training programs might add modules discussing when and how to share ESDs without apologizing for this communication strategy, but also without crossing communication boundaries (McDaniel et al. 2021). Previously, providers in other contexts have described that training about self-disclosure would be useful (Allen and Arroll 2015; Arroll and Allen 2015), but that training is not readily available. ESD guidance in the Dignity Therapy context may be an ideal place to begin this form of provider communication training.

## Limitations and future directions

As this was a first inductive investigation of topics of ESDs shared during Dignity Therapy sessions, we did not apply any sensitizing constructs (e.g., provider gender or discipline, patient illness severity) to draw qualitative comparisons across provider subgroups. We recognize that there may be meaningful differences in patterns of ESDs across provider or patient subgroups that could be examined in future research. This study was also limited by our lack of information from the providers on why they chose to disclose what and when they did. This study focused on transcribed Dignity Therapy sessions themselves, limiting our understanding of providers’ thought processes when engaging in ESD. Interviews with providers about their decisions to disclose in both in-person and videoconferencing Dignity Therapy sessions would allow for a better understanding of these interactions.

Having characterized the types of ESDs made, future research can examine the ways in which different types of ESDs shape patients’ life reviews during Dignity Therapy sessions. We propose 2 pathways by which ESD might shape these conversations. First, certain ESD topics, such as providers’ disclosures of values that align with patients’ values, may help patients feel particularly validated, ultimately leading to patients’ narration of richer life stories. Richer engagement in the Dignity Therapy process may, in turn, promote better outcomes from this intervention (Bluck et al. 2022). In this way, future research should examine patterns of associations between ESD topics, patient communication behaviors, and intervention outcomes. We also propose that different ESD topics may steer the conversation in different ways, with some prompting patients to share details or reflections that would not have sprung to mind had the provider not offered their own disclosure. We recognize that a more in-depth analysis process would be needed to address this possibility. As such, we suggest that future research use discourse analysis techniques (Koenig et al. 2015) to further investigate the impact of providers’ ESDs on Dignity Therapy sessions.

## Conclusions

Self-disclosure made by providers during palliative care patient encounters has received increasing research attention (Rousseau 2009). Dignity Therapy sessions offer unique contexts for studying provider self-disclosure. We demonstrated that providers address or raise various topics when making ESDs, some of which overlap with self-disclosures that are seen as conventional across medical encounter settings (Allen and Arroll 2015). By showcasing the types of ESDs made and exploring when and how ESDs manifest during sessions, our work paves the way for future research to address associations between types of ESDs, communication patterns, and patient outcomes in an advanced cancer context. Further research, combined with our findings, can inform augmentations to training programs for Dignity Therapy and other life review interventions.

## Supporting information

10.1017/S1478951524002098.sm001 Mroz et al. supplementary material Mroz et al. supplementary material

## Supplementary material

The supplementary material for this article can be found at https://doi.org/10.1017/S1478951524002098.
