# Supplementary material for: Sharing “off-script”: A qualitative analysis of providers’ empathic self-disclosures during dignity therapy
Source: Palliat Support Care. 2026 Feb 6;23:e57. doi: 10.1017/S1478951524002098 (PMC11931517; doi:10.1017/S1478951524002098)
Supplement: Mroz et al. supplementary material [file S1478951524002098sup001.docx]

**ECCS DT Coding Manual**

**Empathic Self-Disclosure Code**

Code Definition: The interviewer discloses something substantial and personal about themselves in response to the topic that the interviewee raised. This could be a shared experience, a shared value, or a shared feeling the interviewer seems to be using to relate to the interviewee. If the interviewer discloses something about themselves that is not in response to the interviewee, it would not be counted. (see examples below). One question to ask yourself, did I learn something new about the interviewer personally?

Note to Coders: Please review this and the ARV codebook prior to coding. Also, please listen to the audio recordings while you read the transcripts. This will be helpful in being able to really understand the interaction and the emotions expressed.

Examples of Empathic Self-Disclosure

*Interviewee*: Yeah, just because you’re free. I did the zip lining. I didn’t do it sitting down. I did it flying. I would do that again in a heartbeat.

*Interviewer:* Oh really? I also did zip lining a few years ago – I think I felt something similar to what you said. It felt like I was soaring. I thought it was a lot of fun too. (SHARED EXPERIENCE)

-----

*Interviewee*: […] One of the things that I’d like my kids and my wife to know is that all the prayers for me and all the people who pray for me, it’s humbling *[crying]*. It’s very humbling, *[crying]* to have family and friends like that. *[Crying]*

*Interviewer*: Yeah, I understand how humbling that could be, just knowing how many people care for you and are moved to pray for you. That’s happened to me before too, and it’s always amazing how these moments can happen right when you need them.

(SHARED EXPERIENCE)

-----

*Interviewee*: My kids are my number-one focus right now. In my in life I try to avoid the

mistakes I’ve made, to help raise them basically to do the right thing. I think my dad did a good job trying to his best to raise me, but not really active in my life at a young age. With my kids, I try to be very active in everything they’re doing as far as sports, music. Anything they’re involved in, I want to be there. I think it means a lot to them. I know it does to me.

*[Pause 12:33 – 12:42]*

*Interviewer*: Kids usually appreciate even the smallest of thoughtful acts from their parents. I know when I was little, it meant so much to me when my parents would come to see me sing in the school chorus or even just to take me to the book fairs. (SHARED EXPERIENCE)

-----

*Interviewee:* Dad was tight. He wouldn’t give mom no money. None, no Christmas. He was very tight. I would always give her money, so that’s why we were tight. When I started my own little tile business, I’d give her $100-$200 a week to go to Kmart and hang out, the blue light special—

*Interviewer:* Oh, blue light special. I remember that. (SHARED EXPERIENCE) (implies a similar experience that’s shared amongst interviewee/interviewer)

Note: Generally, responses to direct questions would not be coded as ESD. However, if the interviewer elaborates with content that wasn’t requested in the question or volunteers a shared feeling or experience *empathically*, it would be counted.

----

*Interviewer*: Yeah. It sounds like you had a role as a teacher in a lot of young men’s—a good little man, young men’s lives.
*Interviewee*: Yeah, there was several that I had to show how to do things that I do.
*Interviewer*: Did they know what a nail punch was?
*Interviewee*: Yeah, they knew what a punch was.
*Interviewer*: They knew what a punch was. Well, that’s good so you wasn’t at the very beginning.
*Interviewee*: No, I wasn’t at the very beginning, but it was close. They were just startin’ out.
*Interviewer*: It sounds like you made ‘em want to learn too. (SHARED EXPERIENCE) (implies a similar experience that’s shared amongst interviewee/interviewer)

-----

*Interviewer*: That’s okay. Are there particular things that you feel like you still need to say to your loved ones, things that you have kept to yourself that you feel like you need to say to them?

*Interviewee*: No. Probably, just to encourage them to take care of yourself. Don’t ignore your health. Don’t take it for granted ‘cause my husband end up having to – he was in an accident and had staples put in his head, and he was forced to quit work. I was going through this stuff, disease, and I was forced to quit work. You never know what’s gonna come your way. When they say live your life to the fullest – of course, that’s very hard to do – but just take care of your health. Love your kids. Money’s not the most important thing. Love is. And, I’d say, God. God’s helped a lot.

*Interviewer*: I agree, love is the most important thing, and God is love! I can definitely see His work in your life. (SHARED VALUE)

----

*Interviewee:* My brother and I were able to reconcile about two years ago. Neither of us knew it, but it was just weeks before he died. I am so grateful we had the opportunity. (voice breaks)

*Interviewer:* Wow, I can feel how powerful that was for you. It brought tears to my eyes (SHARED FEELING)

Examples of when you would not code empathic self-disclosure

1. **Not in response to something the interviewee has said.** The following examples would not be coded because they are not in response to something the interviewee has said. In other words, the interviewer is not saying these things as a way to connect with something the interviewee has said about themselves.

*Interviewer*: If we have to backtrack a little, I'll be very sad.

*Interviewee*: That's all right. I can pick it up.

*Interviewer*: I'll wait for … verification.

*Interviewee*: That your bicycle?

*Interviewer*: That one is my spouse's. Mine is underneath it. You can barely see it. Mine is a much slower version. We picked out different kinds. This one is for speed on the streets or somewhere, and mine is for lollygagging with flowers or something.

*Interviewee*: I see. You're the ones that are trying run me down when I'm walking in the forest reserves, right?

*Interviewer*: Not me. Maybe the fast one.

*Interviewee*: Maybe the fast one?

*Interviewer*: I'm a slow lollygagger. I've got a wicker basket on the front.

------

*Interviewee*: Yeah. Could I ask a little about your background, what you—is that off the record?

*Interviewer*: No. You're allowed to. We can let this be the end of the daily therapy. Certainly. We can end our transcription. My background is, I'm—I happen to be a chaplain at [location], and I've been there for about [number] years. By background, I am…

*Interviewee*: Oh, I see.

*Interviewer*: - which is just how that worked. I serve working at the hospital. The executive summary is that I have a deep abiding value of learning people's stories, and so any opportunity that I can sit down to hear them is of deep value, and so this is—it's deeply meaningful. I hesitate to use the word joyful because they're more complicated than that, but it's abidingly meaningful for me to be able to do this.

**The response doesn’t convey empathy**. The response uses self-disclosure but doesn’t empathize with the interviewee’s experience by contributing their own experience. Instead, it contests or tries to reshape the interviewee’s experience by telling their own experience or simply giving their own opinion.

*Interviewee: “when I talked to my oncologist, though, they couldn’t give me a straight answer about my cancer. Like how much time to I really have left?”*

*Interviewer: “Oh, okay. When my mom had cancer, we were glad the doctor didn’t try to tell us how long she had. We were just trying to live in the moment.”*

--------------

*Interviewee: I think she was just a bully. Trying to make my life miserable.*

*Interviewer: You know, I had a friend like that once, but I realized she wasn’t trying to stress me out, she was just going through her own problems. Maybe that’s what your friend was experiencing, too. Not trying to upset you, really.*

*----------------*

*Interviewee: Oh gee. You just hope the world is still here. You hope that life goes back to what it was for them, but I don't know. Yeah, you just—at this point, everything is so uncertain.*

*Interviewer: Okay. Yeah, everything is uncertain right now. Somehow, I suspect it'll come back to some equilibrium, and maybe even improve.*

1. **Agreement that does not convey some sort of personal self-disclosure should not be counted.** Ask yourself “are we learning anything new about the interviewer?”

*Interviewer: What might make ‘em smile? I tell ya something that would make me smile is that hat.*

--------------

*Interviewee: Yeah. There was things that came out durin’ the sermon, and I’ll tell you what some of the notes were. Have hope and faith that not—about my nonbelieving. Durin’ the time I have left, I, personally, am gonna be more focused on God’s purpose for me and my life because, up ’til now—my wife knows I like Frank Sinatra. She believes my theme song, for decades, was I Did It My Way. Now, it’s I do it God’s way.*

*Interviewer: Got it.*

*Interviewee: It makes life a lot easier on everybody.*

*Interviewer: It does.* (Interviewer said this in a very distinct tone).

--------------

*Interviewee: Yeah. You go through stages where you don’t wanna eat and stuff. I do that now with chemo, anyway, for a couple days. Then I’ll eat like a pig. It’s weird. They should’ve took that—remember how they prescribed it back in—remember, there used to be pill mills and stuff?*

*Interviewer: I do remember. Now, let’s go back for a minute to you. I wanna talk a little bit about your brothers. You said you had—you told me yesterday that you have two brothers. One is just a little lazy. He doesn’t come to see your dad as much as—"*

--------------

*Interviewee:* A lot of that, [name], doesn’t come natural. Some of it comes natural because of my personality I was blessed with from God, but a lot of it, you’ve gotta think about. Do you know what the Dale Carnegie course is?

*Interviewer****:*** I do.

--------------

*Interviewee:* Then I went from that school—[school]—to [junior high], but since—middle school then ended in ninth grade, that was just for one year. Then I went to a different school in tenth grade, which was [high school]. [high school] back then still like it is today. I don’t know if you know [high school]. It’s out—

*Interviewer:* I know of it.

--------------

*Interviewee:* It’s just find a relationship with him, get in your word. If you don’t own a Bible anymore, you don’t know where it is, find it, or find it on your phone, and read his word and just see how wonderful he is. We would all love to see our daddy on the other side and myself on the other side, and I just hope that they accomplish that, and we all accomplish that.

*Interviewer:* Yes, absolutely, amen. In creating this record, are there other things you’d like included? Do you want any more about your childhood included or anything?

--------------

Generally, one word type of responses would not count as ESD. E.g, “amen” or “yeah”

--------------

*Interviewee:* I guess just in the end, just remember me. Just remember the good times. If you’re down or whatever, think of somethin’ goofy I did that [laughs] will bring a smile to your face. [Laughs] (voice cracking as she is tearing up)

*Interviewer:* (voice cracking in response, likely tearing up) [Name], that is a beautiful way to end. To offer people smiles and memories. I know I’ve been a stranger with you today, and you allowed me to hear a bit of your story. I wanna say thank you.

--------------
